# Long-term pathogenic response to *Plasmodium relictum* infection in *Culex pipiens* mosquito

**DOI:** 10.1371/journal.pone.0192315

**Published:** 2018-02-05

**Authors:** Romain Pigeault, Manon Villa

**Affiliations:** 1 Department of Ecology and Evolution, University of Lausanne, Lausanne, Switzerland; 2 MIVEGEC, UMR IRD 224-CNRS 5290-Université de Montpellier, Montpellier, France; Université Pierre et Marie Curie, FRANCE

## Abstract

The transmission of *Plasmodium* within a vertebrate host population is strongly associated with the life history traits of its vector. Therefore the effect of malaria infection on mosquito fecundity and longevity has traditionally received a lot of attention. Several species of malaria parasites reduce mosquito fecundity, nevertheless almost all of the studies have focused only on the first gonotrophic cycle. Yet, during their lifetime, female mosquitoes go through several gonotrophic cycles, which raises the question of whether they are able to compensate the fecundity costs induced by the parasite. The impact of *Plasmodium* infection on female longevity is not so clear and has produced conflicting results. Here we measured the impact of *Plasmodium relictum* on its vector’s longevity and fecundity during three consecutive gonotrophic cycles. In accordance with previous studies, we observed a negative impact of *Plasmodium* infection on mosquito (*Culex pipiens*) fecundity in the first gonotrophic cycle. Interestingly, despite having taken two subsequent uninfected blood meals, the negative impact of malaria parasite persisted. Nevertheless no impact of infection on mosquito longevity was observed. Our results are not in line with the hypothesis that the reduction of fecundity observed in infected mosquitoes is an adaptive strategy of *Plasmodium* to increase the longevity of its vector. We discuss the different underlying mechanisms that may explain our results.

## Introduction

The transmission dynamics of malaria parasites within a vertebrate host population is highly associated with the fitness of its vector [1–3]. Consequently, the effect of *Plasmodium* on mosquito fecundity has been widely studied [4–8]. Several species of malaria parasites have been shown to impact negatively the fecundity of mosquitoes [5,9,10]. However, it is often difficult to determine whether this reduction in fecundity is directly induced by the parasite, or whether it is simply a by-product of the lower quality of the infected blood [9,11,12]. Indeed, *Plasmodium* infections induce anaemia in their vertebrate hosts [12,13], and red blood cells are a crucial energy resource for egg production in female mosquitoes [13,14].

Although the Plasmodium effect on fecundity has been studied in many mosquito/parasite systems [9,10,15,16], most articles have focused only on the first *gonotrophic cycle* which is defined as a blood meal followed by an oviposition event [6,7,9–11,17,18]. However, during their lifetime, female mosquitoes take several blood meals, which raises the question of whether they may be able to compensate the fecundity costs induced by an infected blood meal during the following gonotrophic cycles. Whether a single *Plasmodium*-infected blood meal has an impact only on the first gonotrophic cycle, or whether the effect persists for several consecutive gonotrophic cycles can have consequences for the estimation of the lifetime fitness of infected mosquitoes [4], but can also provide insights into the adaptive nature of the fecundity reduction. Indeed, if the fecundity reduction is due to a low quality blood meal (*eg Plasmodium*-triggered anaemia) we may expect it to be more readily compensated for during the following uninfected blood meals, than if it is directly triggered or manipulated by the parasite [19]. Indeed, several studies assumed that the reduction in mosquito fecundity induced by *Plasmodium* infection would be an adaptive strategy of the parasite aimed at increasing mosquito longevity [5,11,20]. This hypothesis is based on the important role of mosquito longevity for *Plasmodium* transmission [8,21].

The specific aim of the present study is thus to determine the impact of *Plasmodium* infection on mosquito (i) longevity and (ii) fecundity during three consecutive gonotrophic cycles (henceforth GC) where only the first blood meal is infected. For this purpose, we have used the avian malaria system consisting of *Plasmodium relictum* and its natural vector in the field *Culex pipiens* [12].

## Methods

### Ethics statement

Animal experiments were carried out in strict accordance with the ‘National Charter on the Ethics of Animal Experimentation’ of the French Government, and all efforts were made to minimize suffering. Experiments were approved by the Ethical Committee for Animal Experimentation under the auspices of the French Ministry of Education and Research (permit number CEEA- LR-1051).

### Biological materials

In Europe *Plasmodium relictum* (lineage SGS1) is the most prevalent form of avian malaria parasite [22]. Avian malaria parasites share a distant common ancestor with human malaria parasites and have historically played an important role as models in the study of human malaria [12]. It is also, to our knowledge, the only experimental (non-human) malaria model that brings together a mosquito-*Plasmodium* combination with a common evolutionary history. The lineage was isolated from infected sparrows in 2009 [12] and passaged to naïve canaries (*Serinus canaria)*. Since then it has been maintained by carrying out regular passages between our stock canaries through intraperitoneal injections [12]. Experimental canaries (n = 2) were infected by injection of 80μl of blood from the infected canary stock. Mosquito blood feeding took place between 10 and 12 days after the injection, using either infected or uninfected birds. Mosquitoes were reared as described by Vézilier *et al*. 2010 [8]. Male and female mosquitoes emerging within a 24h period were provided with 10% sugar solution and kept together, in emergence cages, to allow mating.

### Mosquito fecundity and longevity

**Fig 1:**
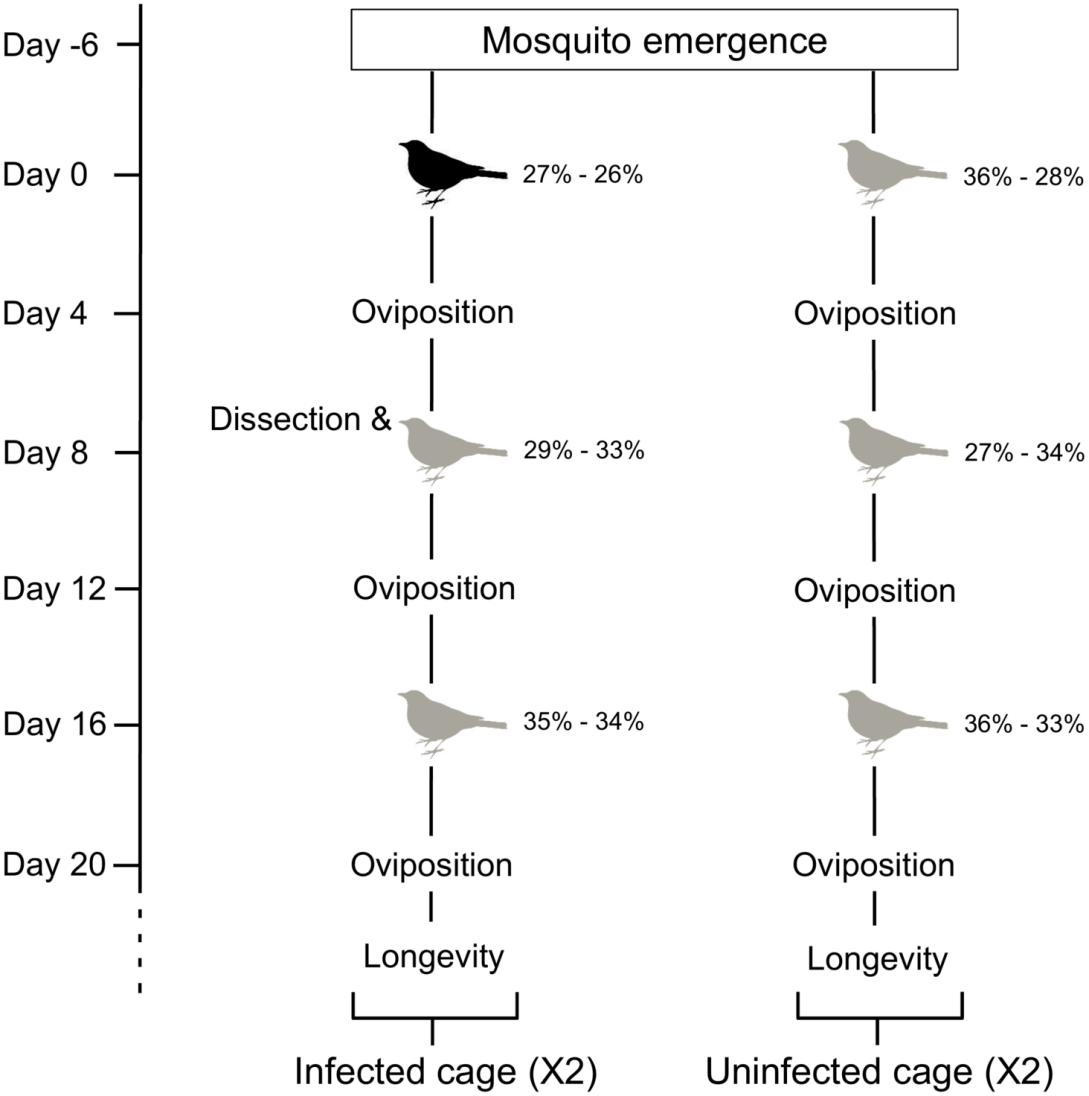
Schematic representation of the experimental design. Grey birds: uninfected blood meal; Black birds: *Plasmodium*-infected blood meal. The percentages, to the right of each birds, correspond to the haematocrit value of the two birds used at each step. *Day* correspond to the day post first blood meal (pfbm). Day 8 pfbm 20 mosquitoes issued from cages containing an infected bird were dissected to count the number of oocysts in their midgut (*Dissection*).

In this experiment, we aimed to explore the effect of *Plasmodium* infection on both fecundity and longevity. The experimental design is shown in Fig 1. Five days after mosquito emergence, mosquitoes were distributed into four cages, two containing 140 females ("infected-cages") and two containing 120 females ("uninfected-cages"). The next day, for the first GC, the two mosquito infected cages were fed on an infected canary and the other two cages with an uninfected one. Immediately after the blood meal, mosquitoes that had not taken a blood meal were counted and removed from the cages. To allow oviposition, 4 days post first blood meal (pfbm), all cages were provided overnight with an oviposition cup, containing mineral water (400ml). The following morning, egg rafts were collected and photographed using a binocular microscope equipped with a camera. The number of eggs per raft were counted using the Mesurim Pro freeware (Academie d’Amiens, France). To obtain an estimate of the infection success, on day 8 pfbm, 20 blood fed females were randomly sampled from both “infected-cages” and killed with CO2. Their midguts were dissected in PBS (standard phosphate-buffered saline) and examined under a microscope to assess oocyst prevalence and burden as in [9]. The same day (8 day pfbm) all cages were provided with a second blood meal followed by an oviposition event (second GC) and, eight days later a third one (third GC). The experimental protocol for the second and third GCs was identical to that described above aside from the fact that all females (for the second and third GC) were fed on uninfected birds (Fig 1).

Mosquitoes were provided with *ad libitum* food in the form of a 10 per cent sugar solution throughout the experiment (excepted during the blood meal period). Longevity of mosquitoes was assessed daily by counting dead individuals lying at the bottom of each cage until all females died.

### Statistical analyses

Analyses were carried out using the R statistical package (v. 3.1.1). The proportion of blood fed females in both mosquito groups was analysed using Pearson’s Chi-squared Test. Data collected on egg production was tested for normality and homoscedasticity using the Shapiro test and Bartlett test respectively. The impact of *Plasmodium* infection and GC on mosquito fecundity (number of eggs laid per female) was analysed using a standard general linear model procedure with a normal error structure. Longevity data were analysed using Cox proportional hazards regression model (coxph, survival package). In this study we are only interested in the impact of *Plasmodium* infection on the longevity of females after the third blood meal (16 day pfbm). For this end, all unfed females were removed and counted after each blood meal.

Maximal models, including all higher order interactions, were simplified by sequentially eliminating non-significant terms and interactions to establish a minimal model [23]. The significant F and χ^2^ values given in the text are for the minimal model, whereas non-significant values correspond to those obtained before the deletion of the variable from the model. Posteriori contrasts were carried out by aggregating factor levels together and by testing the fit of the simplified model using a likelihood-ratio test [23].

## Results

Midgut dissection revealed that almost 95% of the mosquitoes fed on infected bird blood were infected by *Plasmodium relictum* (mean number of oocysts ± s.e. = 84 ± 32). Thereafter, given the very high infection prevalence observed in “infected-cages”, we made the assumption that all egg rafts were laid by infected mosquitoes.

In both experimental groups (“infected” and “uninfected-cages”), the proportion of females which took a blood meal at each GC were not statistically different (1st GC: χ^2^_1_ = 0.448 p = 0.503, 2nd GC: χ^2^_1_ = 1.784 p = 0.282 and 3rd GC: χ^2^_1_ = 2.010 p = 0.1562, S1 Table).

**Fig 2:**
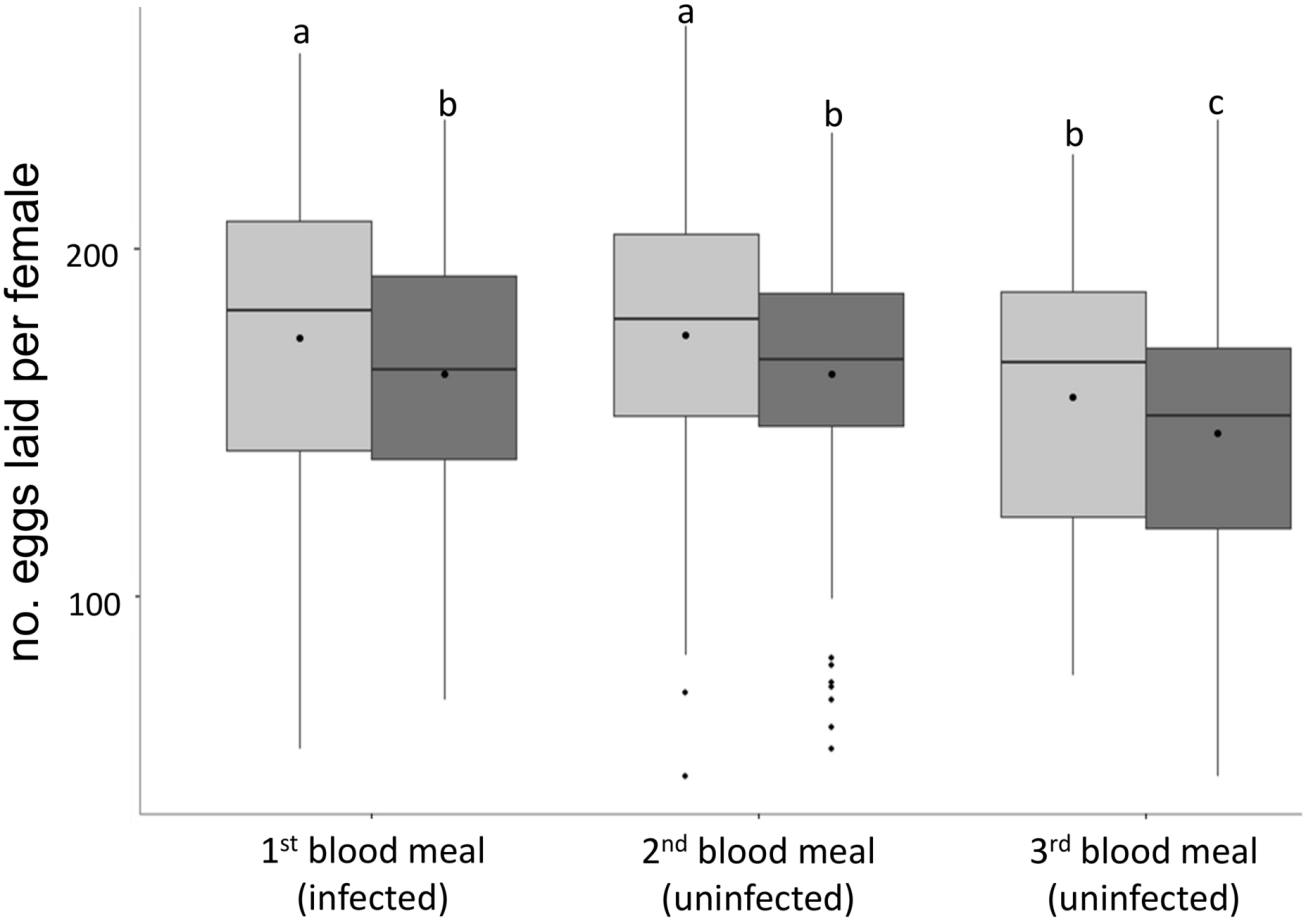
Impact of *Plasmodium* infection on the number of eggs laid by mosquitoes during three consecutive gonotrophic cycles. Boxplots of the number of eggs laid per female fed on control uninfected (light grey boxes) or *Plasmodium* -infected birds (dark grey boxes). Only the first blood meal was infected by *P*. *relictum* (in the case of the dark box). Boxplots represent the means (points) and medians (horizontal lines). Boxes above and below the medians show the first and third quartiles respectively, lines delimit 1.5 times the inter-quartile range, above which individual counts are considered outliers and marked as full circles Different letters (above boxplots) indicate significant differences between the number of eggs laid by different mosquito group based on contrast analysis test p < 0.05.

**Fig 3:**
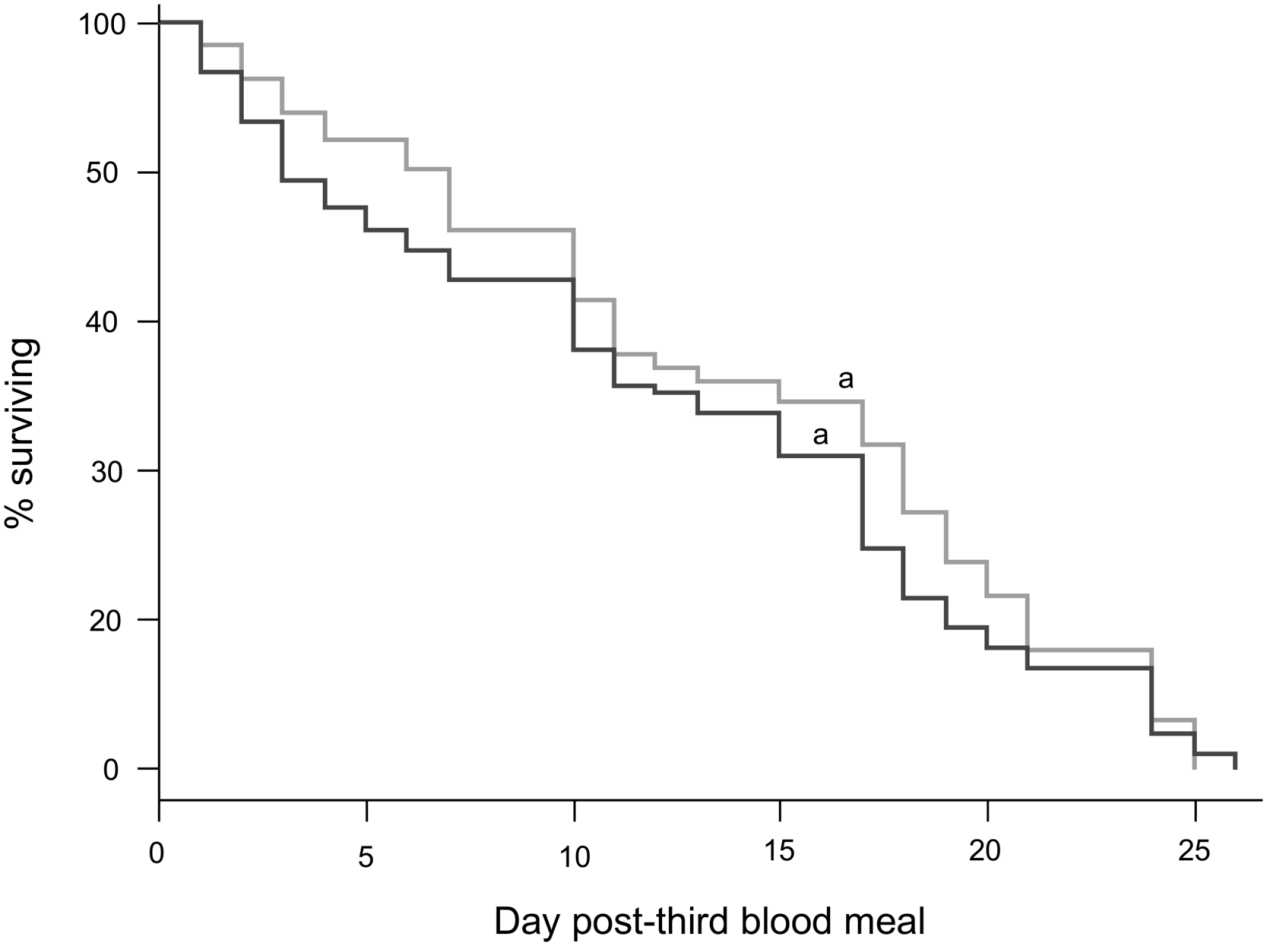
Mosquito survivorship after the third blood meal. Kaplan—Meier survival curve for the uninfected (grey) and infected mosquitoes (dark grey). Curves not connected by same letter are significantly different.

GC and *Plasmodium* infection both had an impact on mosquito fecundity (number of eggs laid by females, respectively F_2,692_ = 11.855, p < 0.0001, F_1,694_ = 12.062, p < 0.001, Fig 2). In all GCs all females which were initially provided with an infected blood meal laid significantly fewer eggs than control females (Fig 2). There were no significant differences in the number of eggs laid in the 1st and second GCs by either uninfected (contrast analyses 1st vs 2nd GC: χ^2^_1_ = 0.163 p = 0.923) or infected (χ^2^_1_ = 0.047 p = 0.953) females (Fig 2). Fecundity, however, decreases between the 2nd and 3rd GCs for both uninfected (contrast analyses: χ^2^_1_ = 16.225 p = 0.009) and infected females (contrast analyses: χ^2^_1_ = 19.825 p < 0.001). It should be noted that fecundity differences observed between infected and uninfected mosquito, fed on uninfected birds (2nd and 3rd GC), do not seem to be explained by differences in hosts’ hematocryte (Fig 1). Indeed, hosts’ hematocryte were similar (Fig 1). The longevity of females after the third blood meal was not impacted by *Plasmodium* infection (χ^2^_1_ = 1.161 p = 0.281, Fig 3, S1 Table).

## Discussion

In accordance with previous studies in several malaria systems [9,10,15,16] but see [18], we observed a negative impact of *Plasmodium* on mosquito fecundity in the first GC following an infected blood meal. However, the cost payed by infected females was relatively low (Fig 2). This can be explained in that we used a *Plasmodium* strain maintained under laboratory condition for 4 years. Indeed, in a recent study we shown that the serial passages of this strain had selected for higher parasitaemia in the vertebrate host [12] but also for lower cost in mosquitoes (Pigeault *et al*. 2016 unpublished). However, despite having taken two subsequent uninfected blood meals, the negative impact of *Plasmodium* infection persisted through to the third GC. Similar results have been observed by Hogg & Hurd [4] albeit using an artificial combination between parasite and vector.

There are several potential explanations for the differences in fecundity observed between infected and uninfected mosquitoes through several consecutive GCs. First, fecundity is known to be strongly correlated with the blood meal size [6,12]. Although the reduction of female fecundity observed after an infected blood meal cannot be only explained by a smaller quantity of blood ingested [5], infected females could take smaller blood meals subsequently. This could happen if the damage to the midgut epithelium caused by the presence of oocysts [24] reduces the efficiency of blood digestion. Unfortunately, in our experiment, haematin production was not quantified and thus this hypothesis could not be tested. However, work carried out in other systems has shown that blood meal size is not affected by the presence of either midgut oocysts [4] or sporozoites [25] in the salivary glands, rendering this explanation unlikely. Second, vertebrate blood quality is also important for mosquito fecundity [14]. Malaria parasites decrease red blood cell density, glucose, lipid, aminoacid and micronutrient composition of host blood [26], either because these nutrients are used by the parasite or by the host during the response to the infection. Under this scenario, the negative effects of a low quality (infected) blood meal on mosquito fecundity may not have been compensated for by two subsequent healthy blood meals. The limited work available, however, suggests that in *Culex quinquefaciatus* the quality of the first blood meal does not have carry-over effects on the second blood meal [27]. Third, trade-offs between immunity and reproduction could also explain the persistent cost of *Plasmodium* infection [28]. Indeed, the second and third GCs take place at the same time as oocysts and sporozoites, respectively, from the first GC reach their peak [9], and when activation of the mosquito immune response may be maximal. The activation of the immune response in mosquitoes has been shown to induce a strong cost on female fecundity [29] but the dynamics of the mosquito’s immune response after *Plasmodium* infection remains poorly understood. Finally, the pervasive effects of a *Plasmodium* infection on mosquito fecundity could be the direct effect of the parasite on mosquito physiology. Indeed, the reduction of fecundity observed in infected mosquitoes is explained in part by both a reduction of yolk protein provide by the ovaries and by an increase in egg resorption caused by follicular cells apoptosis [5,30]. Nevertheless, studies showed that the effect of *Plasmodium* infection is not associated to the oocysts burden [4,6]. The presence of the parasite would act as an ON / OFF button. Indeed, once females are infected, regardless the parasite load, a mechanism impacts the oogenesis [5,30]. This reduction in mosquito fecundity could be an adaptive strategy of the parasite to increase the longevity of its vector through a trade-off in resource allocation between reproduction and longevity [9,31]. Indeed, a study has shown that a reduction in fecundity in *Cx*. *pipiens* mosquito infected by *P*. *relictum* during their first GC is accompanied by a significant increase in longevity [9].

Nevertheless, in our study we did not observe any effect of *P*. *relictum* infection on mosquito longevity after the third GC. To date, the impact of *Plasmodium* infection on mosquito longevity has produced conflicting results [9,32,33,34]. The effect of malaria infection on mosquito longevity seems to be extremely sensitive to the particular mosquito-*Plasmodium* combination used and to the specific laboratory conditions under which it is measured [9,32,33,34]. For instance, a negative effect of malaria infection on mosquito longevity was observed under nutritional stress (low glucose conditions [33]) or when mosquitoes did not have the opportunity to lay eggs [34]. Here, mosquitoes were allowed to feed *ad libitum* (10% sugar solution) and females laid eggs after each blood meal session. In this experimental conditions and after three gonotrophic cycles we did not observe any effect of *P*. *relictum* infection on Cx *pipiens* longevity. To our knowledge, so far the majority of studies have focused on the impact of *Plasmodium* infection on mosquito longevity during only one gonotrophic cycle. Multiple blood meals, after an infected one, could potentially limit or decrease the effect of *Plasmodium* infection on the longevity of its vector. Indeed, multiple blood-feeding may impact directly the longevity of mosquito [35]. The effect of *Plasmodium* infection on the longevity of its vector requires further investigation, because of its potential epidemiological consequences [9,21].

### Conclusions

The negative impact of *P*. *relictum* on Cx *pipiens* fecundity is not dependent on the parasite burden [6] and persists for, at least, three consecutive gonotrophic cycles. Hurd and Hogg [14] obtained similar results with *An*. *stephensi* infected with the rodent malaria parasite *P*. *yoelii* but tempered their conclusions by suggesting that they may have been an artefact of an unnatural parasite-mosquito combination. Our results show that these effects are also present in parasite-mosquito combinations found in nature. Nevertheless, no effect of infection on vector longevity was observed. Our results thus show that the long-term pathogenic response to *Plasmodium* infection in mosquito seems not to be a parasite adaptive strategy to increase mosquito survival.

## Supporting information

S1 Table Effect of *Plasmodium* infection and gonotrophic cycle on blood meal rate, laying rate and longevity of mosquito. *Total alive*: number of females in the cage during the blood meal session. *Blood fed*: Number of blood fed females after the blood meal session. *Unfed*: Number of unfed females after the blood meal session. *nb of egg raft laid*: Number of egg raft laid after the blood meal session. All unfed female were immediately removed after each blood meal session. The number of females alive in the next blood meal session is the number of blood fed females in the previous blood meal session minus the number of dead females.
(DOCX)

S1 Dataset Data. Dataset providing fecundity (number of eggs) and longevity data of Cx *pipiens* mosquito exposed or not to *Plasmodium relictum* infection.
(XLSX)
