# Supplementary material for: Long-term pathogenic response to Plasmodium relictum infection in Culex pipiens mosquito
Source: PLoS One. 2018 Feb 5;13(2):e0192315. doi: 10.1371/journal.pone.0192315 (PMC5798818; doi:10.1371/journal.pone.0192315)
Supplement: S1 Table — Total alive: number of females in the cage during the blood meal session. Blood fed: Number of blood fed females after the blood meal session. Unfed: Number of unfed females after the blood meal session. nb of egg raft laid: Number of egg raft laid after the blood meal session. All unfed female were immediately removed after each blood meal session. The number of females alive in the next blood meal session is the number of blood fed females in the previous blood meal session minus the number of dead females. (DOCX) [file pone.0192315.s001.docx]

Long-term pathogenic response to *Plasmodium relictum* infection in *Culex pipiens* mosquito

Romain Pigeault^12¶*^, Manon Villa^2¶^

**Table S1: Effect of *Plasmodium* infection and gonotrophic cycle on blood meal rate, laying rate and longevity of mosquito.**

**Table S1: Effect of *Plasmodium* infection and gonotrophic cycle on blood meal rate, laying rate and longevity of mosquito.** *Total alive*: number of females in the cage during the blood meal session. *Blood fed*: Number of blood fed females after the blood meal session. *Unfed*: Number of unfed females after the blood meal session. *nb of egg raft laid* : Number of egg raft laid after the blood meal session. All unfed female were immediately removed after each blood meal session. The number of females alive in the next blood meal session is the number of blood fed females in the previous blood meal session minus the number of dead females.

|  | **Infected group** | | | | **Uninfected group** | | | |
| --- | --- | --- | --- | --- | --- | --- | --- | --- |
|  | Total alive | Blood fed | Unfed | nb of egg raft laid | Total alive | Blood fed | Unfed | nb of egg raft laid |
| 1st blood meal | 280 | 192 | 88 | 189 | 240 | 157 | 83 | 133 |
| 2nd blood meal | 171 | 141 | 30 | 134 | 153 | 116 | 37 | 117 |
| 3rd blood meal | 122 | 108 | 14 | 90 | 111 | 105 | 6 | 82 |
